# Supplementary material for: Genomic epidemiology of Enterococcus faecium bloodstream infections during a VanB-type VRE peak reveals an oligoclonal scenario: an observational study at a German university hospital (2017–2022)
Source: J Clin Microbiol. 2026 Mar 18;64(4):e01044-25. doi: 10.1128/jcm.01044-25 (PMC13059727; doi:10.1128/jcm.01044-25)

Supplement to

Genomic Epidemiology of *Enterococcus faecium* Bloodstream Infections During a VanB-type VRE Peak Reveals an Oligoclonal Scenario: An Observational Study at a German University Hospital (2017–2022)

Leonard Knegendorf & Anna Sommer, Claas Baier, Robert E. Weber, Martin A. Fischer, Guido Werner, Stefan Ziesing, Dirk Schlüter

SUPPLEMENTARY FIGURE 1. Heatmap of virulence factors in the most prevalent complex types (CTs). Columns represent virulence genes and variant calls as defined by *VirulenceFinder* (Center for Genomic Epidemiology, Technical University of Denmark). Each row corresponds to one of the most prevalent sequence type (ST)-CT combinations identified in this study. Color intensity indicates the percentage (0–100%) of isolates carrying each variant, with darker shades representing higher percentages. If an isolate matched more than one variant in the database with identical identity and gene coverage, this was defined as an *ambiguous call*. In the heatmap, only the percentage for the dominant variant within each CT is shown. Abbreviations: Efm, *E. faecium*; Elts, *E. lactis*; PGC, pili gene cluster; PTS, phosphotransferase system.

SUPPLEMENTARY FIGURE 2. Timeline of the VRE BSI cases assigned to cgMLST cluster 2. Each row represents a single patient (n=47), grouped by the treating specialty. These specialties were categorized into three organ system–related groups (see Materials and Methods). The timeline spans the whole study period (monthly ticks). Patients with a blue-colored patient identifier had a known intestinal VRE colonization prior to or at the time of BSI. The SKA-Cluster column lists the SKA clusters (11-18) assigned to each patient's isolate(s). Colored boxes (or narrow vertical bars for short stays) indicate the hospital ward(s) where each patient was admitted during the corresponding time period; each color denotes a distinct ward. The letter "i" marks the date the VRE-positive blood culture was obtained.

Supplementary Figure 1

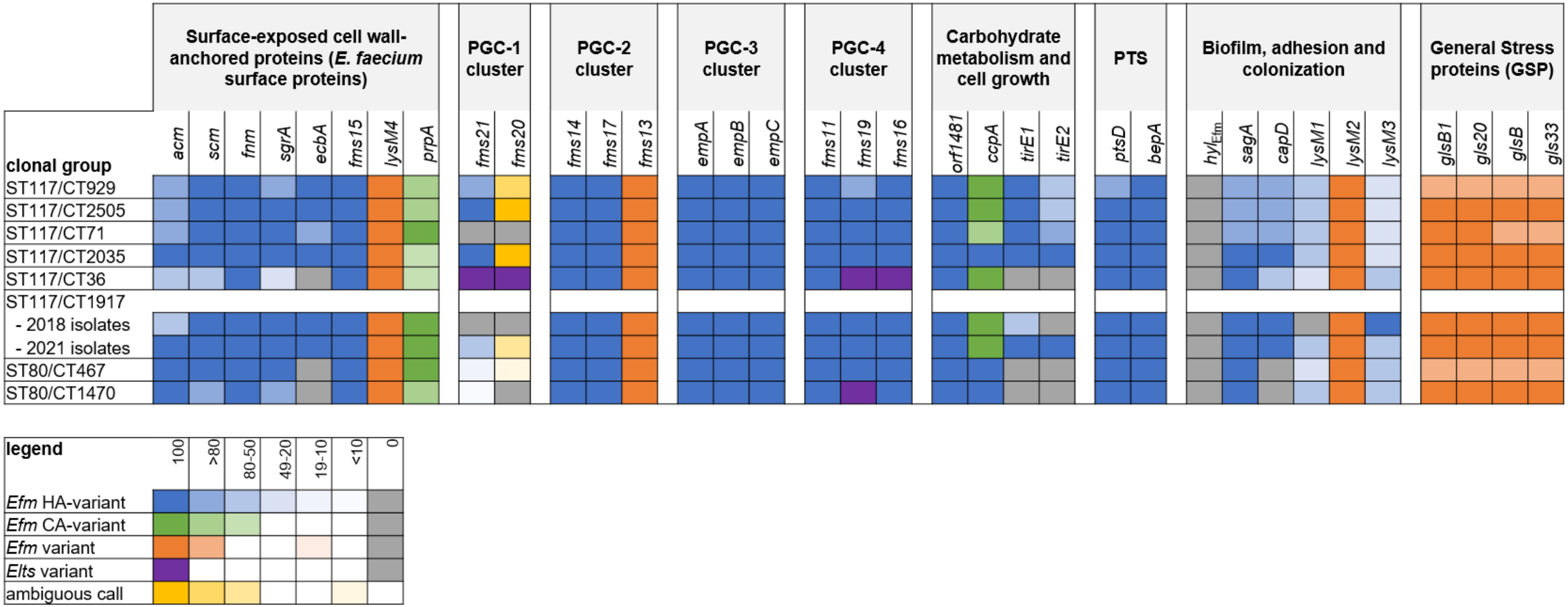

Supplementary Figure 2

Underlying disease (organ system)	Patient pseudonym	SKA-Cluster
Excretory system	12346	
Excretory system	11702	
Excretory system	10985	14
Excretory system	10158	
Excretory system	10860	
Excretory system	11793	
Excretory system	11668	11
Excretory system	13067	
Excretory system	13269	12
Excretory system	10113	
Excretory system	11675	12
Excretory system	13490	
Excretory system	12325	
Excretory system	11149	11
Excretory system	12304	13
Excretory system	10716	13
Excretory system	12636	17
Excretory system	10466	
Excretory system	11894	
Excretory system	12650	
Hematopoietic system	11171	
Hematopoietic system	12037	14
Hematopoietic system	10701	14
Hematopoietic system	12982	15
Hematopoietic system	11810	15
Hematopoietic system	10278	
Hematopoietic system	12711	
Hematopoietic system	10271	16
Hematopoietic system	12641	16
Hematopoietic system	11327	
Hematopoietic system	12907	18
Hematopoietic system	12331	
Hematopoietic system	12712	
Hematopoietic system	12256	
Other	10566	
Respiratory and cardiovascular system	12875	13
Respiratory and cardiovascular system	13122	
Respiratory and cardiovascular system	12229	
Respiratory and cardiovascular system	11304	12
Respiratory and cardiovascular system	13007	
Respiratory and cardiovascular system	11002	
Respiratory and cardiovascular system	11089	11
Respiratory and cardiovascular system	12854	
Respiratory and cardiovascular system	11311	
Respiratory and cardiovascular system	12457	
Respiratory and cardiovascular system	11830	
Respiratory and cardiovascular system	12916	

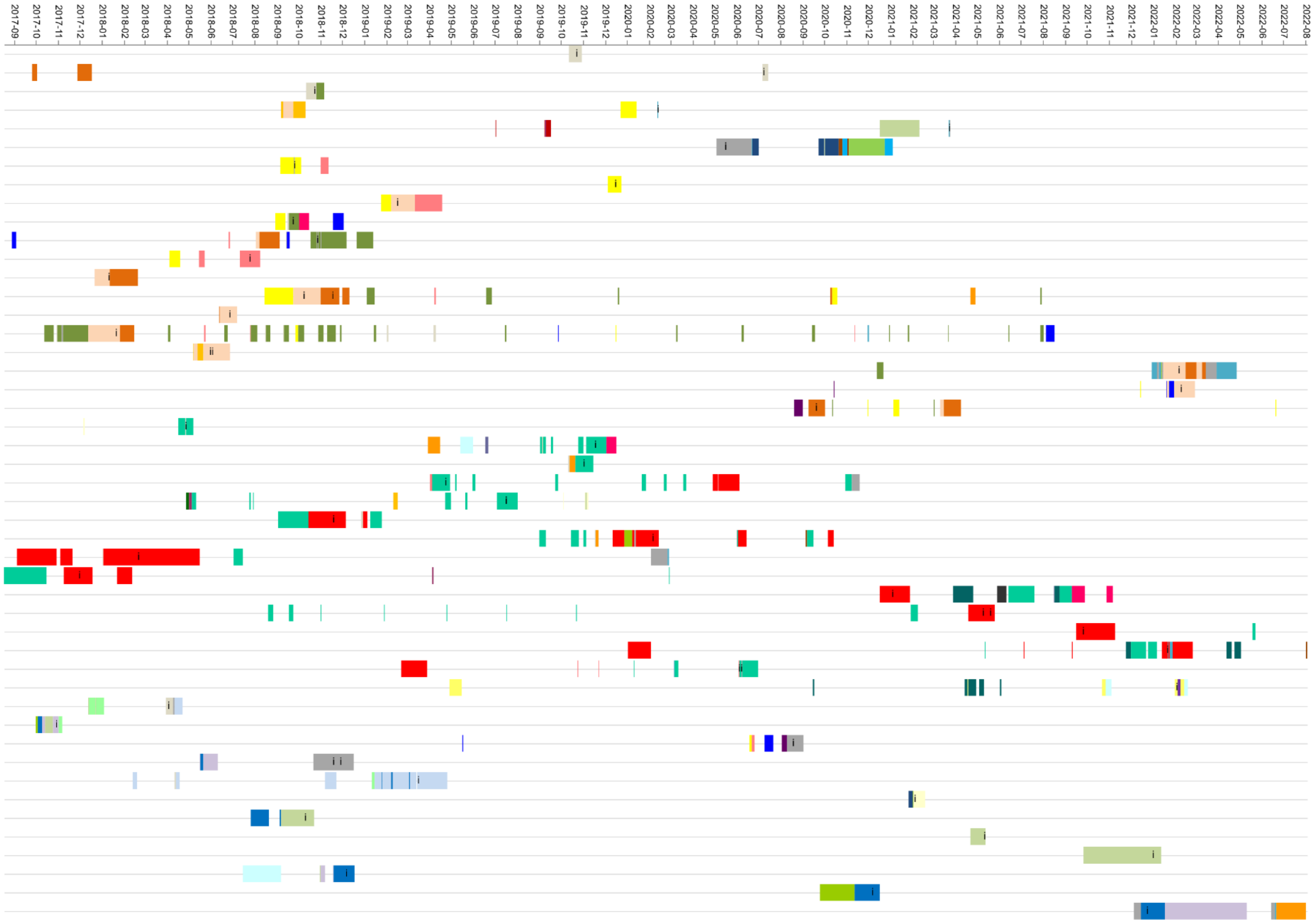

Supplement: Fig. S1 and S2 — VirulenceFinder results for major clonal lineages and timeline of the VRE BSI cases assigned to cgMLST cluster 2. [file jcm.01044-25-s0001.pdf]
